# Space Flight Enhances Stress Pathways in Human Neural Stem Cells

**DOI:** 10.3390/biom14010065

**Published:** 2024-01-03

**Authors:** Nicholas Carpo, Victoria Tran, Juan Carlos Biancotti, Carlos Cepeda, Araceli Espinosa-Jeffrey

**Affiliations:** 1 Department of Psychiatry, UCLA, Los Angeles, CA 90095, USAvictoriakimtran@gmail.com (V.T.); ccepeda@mednet.ucla.edu (C.C.); 2 Department of Surgery, Division of Pediatric Surgery, School of Medicine, Johns Hopkins University, Baltimore, MD 21205, USA; jbianco5@jh.edu

**Keywords:** microgravity, space flight, human neural stem cells, cell stress, intracranial hypertension

## Abstract

Mammalian cells have evolved to function under Earth’s gravity, but how they respond to microgravity remains largely unknown. Neural stem cells (NSCs) are essential for the maintenance of central nervous system (CNS) functions during development and the regeneration of all CNS cell populations. Here, we examined the behavior of space (SPC)-flown NSCs as they readapted to Earth’s gravity. We found that most of these cells survived the space flight and self-renewed. Yet, some showed enhanced stress responses as well as autophagy-like behavior. To ascertain if the secretome from SPC-flown NSCs contained molecules inducing these responses, we incubated naïve, non-starved NSCs in a medium containing SPC-NSC secretome. We found a four-fold increase in stress responses. Proteomic analysis of the secretome revealed that the protein of the highest content produced by SPC-NSCs was secreted protein acidic and rich in cysteine (SPARC), which induces endoplasmic reticulum (ER) stress, resulting in the cell’s demise. These results offer novel knowledge on the response of neural cells, particularly NSCs, subjected to space microgravity. Moreover, some secreted proteins have been identified as microgravity sensing, paving a new venue for future research aiming at targeting the SPARC metabolism. Although we did not establish a direct relationship between microgravity-induced stress and SPARC as a potential marker, these results represent the first step in the identification of gravity sensing molecules as targets to be modulated and to design effective countermeasures to mitigate intracranial hypertension in astronauts using structure-based protein design.

## 1. Introduction

After space flight, health anomalies, such as intracranial hypertension, flattening of the rear of the eyeball, bulging of the optic nerve, and other anatomical and systemic alterations such as white matter microstructural changes, have been reported [1,2]. Therefore, microgravity-induced intracranial hypertension represents a potential limitation for long-duration space flight. Neural stem cells (NSCs) are crucial in the central nervous system (CNS) because they self-renew and also give rise to neurons, astrocytes, and oligodendrocytes. Moreover, they maintain the integrity and function of the CNS. Previously, we demonstrated that, just like in simulated microgravity, space microgravity (SPC-µG) induces enhanced proliferation of NSCs. We corroborated this during space flight [3] and confirmed that NSCs proliferated more than ground controls after space flight [4]. We also found that the vast majority of these cells adapted to Earth’s gravity and displayed typical features of NSCs, such as self-renewal via normal proliferation, where cytokinesis occurred within the range of ground control cells without significant differences [4]. In this study, we examined the behavior of space-flown NSCs as they were adapting to Earth’s gravity in more detail and found that some of the SPC-flown NSCs and their progenies displayed autophagy-related events likely caused by endoplasmic reticulum (ER) stress. For the purpose of this paper, we named this phenomenon “autophagy-like behavior” (ALB). This phenomenon is reminiscent of what occurs during the development of the CNS. Nonetheless, it also participates in cell survival and cell death [5]. To examine if the secretome from SPC-flown NSCs contained a molecule(s) that would induce ALB, we cultured naïve NSCs in non-starving conditions using fresh NSC growth medium containing the secretome produced by NSCs flown to space. There was a four-fold increase in ALB rate compared to non-treated naïve NSCs, raising the imminent need to analyze the secretome contents of SPC-flown cells to start unraveling what had enhanced this behavior. We report that the protein with the highest content was SPARC. This protein has been attributed numerous functions, it modulates the endothelial barrier function [6], it is constitutively expressed by proliferative human cerebral microvascular endothelial cells (hCMEC/D3), and its expression declines as these cells mature [6]. The encoded protein is required for collagen in the bone to become calcified. It is also involved in extracellular matrix synthesis [7] and induces cellular morphological changes. Among its pleiotropic functions, autophagy supports cell survival as well as cell death in different cells, organs, and conditions [8,9].

Altogether, our work uncovers the response of human neural cells, particularly NSCs, to the gravitational regulation of several stress-related proteins. Moreover, it reveals an important phenomenon of the memory pool wherein some daughter NSCs born to SPC-flown ones display ALB under Earth’s gravity. These novel features would not be possible to find in studies performed solely under Earth’s gravity because SPARC is upregulated by space microgravity. Moreover, this study is significant to astronauts’ health because it may be enhanced as a mechanism to compensate for the increased number of NSCs produced during and after space flight [3,4], which may induce a limited expansion of the astronauts’ total brain size.

## 2. Materials and Methods

### 2.1. Cells and Culture System

Human tissue experiments were approved by the Office of the Human Subject Committee. Specimens donated by the Department of Pathology and Laboratory of Medicine at the University of California, Los Angeles (UCLA), were the source of the cells used for this study.

Samples were de-identified prior to their use in accordance with National Institutes of Health (NIH) guidelines. Anonymous, preserved specimens are donated for medical research purposes and are Institutional Review Board (IRB) exempt (www.pathology.ucla.edu/TPCL.html).

### 2.2. Cultures of NSCs

A population of NSCs was obtained from human-induced pluripotent stem cells (hiPS) in Cedars-Sinai Medical Center. The original cells, known as “CS83iCTR-33n1” (derived from skin cells), were “reprogrammed”. We then induced these hiPS to the neural phenotype using our stem cell medium (STM). For detailed information on the culture medium and cell preparation, see previous work [10,11,12].

### 2.3. Space Flight

The BioScience-4 mission was launched onboard the Space-X 16 Dragon capsule on 5 November 2018, being, to the best of our knowledge, the first study to investigate the behavior of human CNS stem cells in space microgravity and upon their return to Earth. This study contributes to a better understanding of changes produced in neural cells while in space, as well as after returning to Earth [3,13]. For space flight, NSCs were seeded onto 8-well hardware (Yuri), then flown to the ISS and installed in the Space Technology and Advanced Research System Experiment Facility-1 (STARS-F1, STaARS) at 37 °C. Cells remained onboard the International Space Station (ISS) for 39.3 days and then returned to Earth.

### 2.4. Recovery of the Hardware and Harvesting of Samples

**Figure 1:**
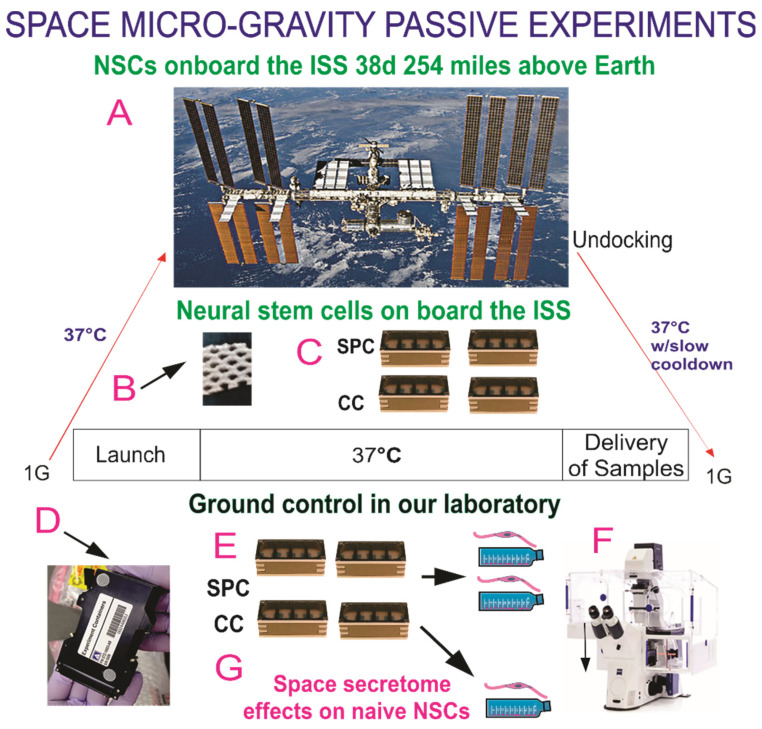
Human neural stem cells (hNSCs) were flown to the International Space Station (ISS) on board SpaceX-16 as part of the Bioscience-4 Space Biology NASA experiment. It launched on 5 November 2018 and landed (splash-down) on 15 January 2019. On the ISS, astronauts receive about 150 mGV per six months. The average daily total radiation dose for the current mission was 0.425 mGv on board the space station. (**A**) View of the ISS. The cells remained in microgravity for 39.3 days. We designed this experiment to mimic the trajectory astronauts undergo during space flight (i.e., launch, stay in space, and splash down when returning to Earth) without intervention of astronauts or specialized hardware for medium change. (**B**) View of the mesh carrier prior to seeding NSCs onto it. (**C**) View of the four 8-well units used for the passive experiments on board the ISS. (**D**) External cover in which 8-well chambers traveled. (**E**) View of the external shell in which the units travelled and stayed while in space. The same number of chambers were flown to space and left on Earth in our laboratory as ground control. All cells were maintained at 37 °C. Ground control cells were seeded in similar containers and maintained at the same temperature and conditions in our laboratory; the only difference between the set of NSCs flown to the ISS and the ground control set that remained in our laboratory was that the former was subjected to microgravity during space flight while the latter was subjected to only gravity. (**F**) Upon splashdown, all samples were transferred from Kennedy Space Center to World Carrier and brought to Long Beach CA. They were transported to our laboratory (UCLA) at 37 °C. After recovery from the hardware, NSCs were seeded in fresh medium for time-lapse studies. (**G**) The secretome was collected from each well separately.

After splashdown, samples were transported in a controlled environment from Long Beach airport to UCLA. Subsequently, NSCs were recovered from each well separately. The secretome was frozen for further use. NSCs were retrieved from the hardware, plated onto poly-d-lysine coated flasks in STM [10], and allowed to recover from space flight. After 20 h in the incubator (5% CO_2_ and at 36.8 °C), flasks were placed in a Zeiss Axio Observer 7 to visualize cells as they were adapting to terrestrial gravity (Figure 1).

### 2.5. Time-Lapse Microscopy

A Zeiss Axio Observer 7 fully motorized inverted research microscope was used. It was equipped with the Zeiss Axiocam 506 monochrome camera. The system was equipped with the full Incubation XL chamber (Zeiss, Jena, Germany) for temperature control and with a motorized scanning stage. Zeiss ZEN 3.0 software and definite focus were essential for this study.

Images are displayed at 4 frames per second, where frames represent image captures taken 10 min apart.

### 2.6. Cell Counts and Statistical Analysis

Statistical analyses were performed using One-Way ANOVA, followed by Tukey post hoc test when multiple groups were compared in which *p* < 0.05 was defined as statistically significant. For two group comparisons, we used Student’s *t*-test, in which *p* < 0.05 was defined as statistically significant. Statistical data are presented as mean ± SD.

### 2.7. Secretome Collection and Proteomics Analysis

We used automated hardware (Yuri) that allowed us to collect the medium produced solely in space during 26 h as previously described [12]. A synopsis of the experiment is shown in Figure S1. The culture medium that fed the cells during space flight was recovered from the hardware separately. The media from the cell chamber and from each tank were placed in numbered tubes with addition of proteases inhibitor cocktail, and saved frozen at −80 °C. For the purpose of this paper, we named this conditioned medium “secretome”.

#### Two-Dimensional DIGE Preparation of Samples and Proteomic Analysis

The conditioned medium samples (secretome) were thawed and vortexed for 20 s. The samples were spun for 30 min at 4 °C at 14,000 rpm and the supernatant was collected. The detailed methods have been previously described [14].

## 3. Results

### 3.1. Post-Space Flight Observations of NSCs

In the current study, we were not able to visualize NSCs while in space due to lack of astronaut-time and the type of hardware chosen. Nonetheless, post-flight observations using time-lapse microscopy allowed us to follow their behavior and recovery in detail. After harvesting them from the 8-well flown hardware, NSCs were seeded onto poly-d-lysine coated flaskettes, with glass bottoms for optimal quality imaging.

### 3.2. Space Flight Activates Stress Responses and ALB in NSCs

**Figure 2:**
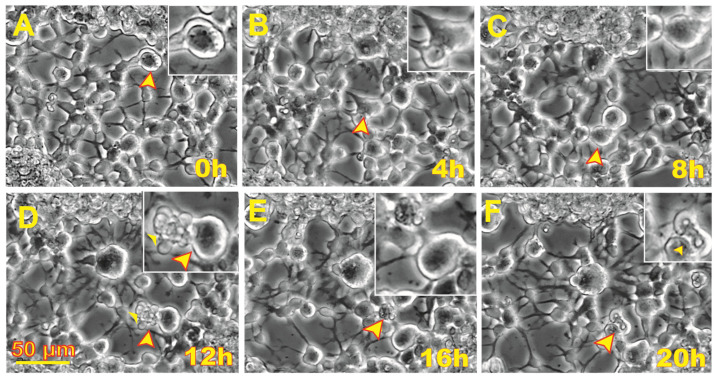
Human neural stem cells (NSCs) exhibited autophagy-like behavior (ALB) after space flight. Sequential views of the behavior of an NSC flown to space one week after returning to Earth. These views were analyzed as cells with ALB were counted and evaluated through tracking in reverse time. (**A**) Cell 13.25 h following initiation of the time-lapse capture. (**B**) The cell shows structural transformations as it appears to fuse with another NSC. (**C**) After 8 h following the initial frame, the cell underwent morphological differences again and had not fused with its neighbor. (**D**) After 4 h, some cells’ contents were expelled. (**E**) After another 4 h, the cell had internalized the contents which were previously expelled. (**F**) Then, a pocket-like formation formed through the contraction of the cell within 20 h. A video of this event is shown in Video S1.

**Figure 3:**
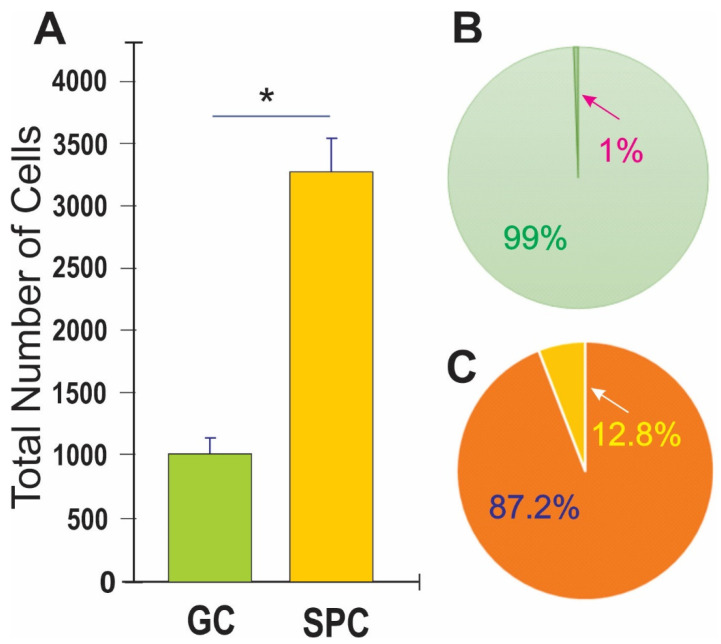
Human NSCs displayed enhanced ALB after space flight. (**A**) This bar graph shows the average of the total number of cells present 44 h after the time-lapse started. The green bar denotes ground control (GC)-NSCs. The orange bar denotes SPC-NSCs one week post-flight. (**B**) Results shown as a percentage where only 1% of GC-NSCs exhibited ALB (green pie graph) as compared to the 12% SPC-NSCs showing ALB orange pie graph. (**C**) Data in A were compared using Student’s *t* test, in which * *p* < 0.05 was defined as statistically significant. Data represent the mean of four separate scenes for each color-coded condition.

After seeding onto flaskettes, NSCs were placed in the time-lapse microscope system We captured phase contrast images every 10 min to visualize the changes occurring in cells after space flight. We found that, compared to controls, a higher percentage of SPC-flown NSCs displayed ALB (Figure 2 and Figure 3).

### 3.3. SPC-Flown NSCs’ Secretome Increases Naïve NSCs ALB

**Figure 4:**
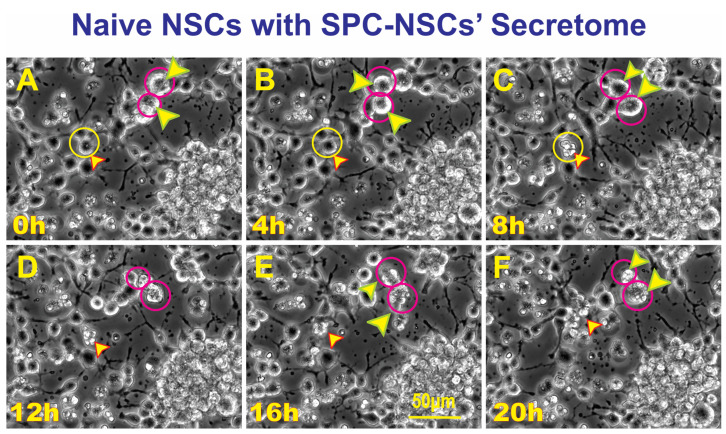
Unveiled effects of SPC-NSC-secretome on enhanced ALB in naïve NSCs. Sequential views of the behavior of naïve NSCs incubated in a culture medium containing the secretome produced by NSCs flown to space. The cultures were examined to identify cells undergoing ALB. Once a cell was identified, it was then studied through reverse-time tracking. At the beginning of the time-lapse acquisition, the cells looked round and healthy. Note that at least three instances of ALB are observed in the field of view. (**A**) View of a cell of interest 13.25 h after the beginning of the time-lapse study. Yellow circle and arrow, in the same field two larger cells labeled with red circles and arrows. (**B**) Morphological changes observed, the cell encircled in yellow looks healthy and still bearing cell processes. The two larger cells encircled in red look rounder and healthy as well; (**C**) after 8 h following the start of the time-lapse, the cell had expelled material from its soma (yellow circle). The two cells inside red circles were less round and still looked healthy. (**D**) In the span of four hours, the cell in the top red circle had expelled some of its contents onto the extracellular space while the one in the bottom circle looked ruffled. (**E**) Another 4 h later, the cell in the top red circle appears to have internalized the expelled material, while the cell below that one had become larger. (**F**) The cell then collapsed and became just a small pocket-like structure. The total duration of this event was 20 h. Video time-lapse of this event is shown in Video S3.

Having observed ALB in some of the NSCs back from space, we then sought to determine if naïve NSCs that had not been left unattended and were fed fresh culture medium prior to performing the experiment would respond to the secretome from SPC-flown NSCs by displaying autophagic behavior. Naïve NSCs were plated onto the flaskettes (flasks on slide) and left overnight in STMc. The next day, to make sure that cells were not starved, fresh STMc medium with SPC-secretome (2:1) was prepared to replace the medium in the culture to be imaged. Interestingly, upon exposure to the SPC-secretome, we observed the same phenomenon, displayed by SPC-NSCs and their progenies, in naïve NSCs. The response was gradual and increased with time after incubation in the SPC-secretome-supplemented culture medium (Figure 4). A video time-lapse of this event is shown in Video S2.

### 3.4. ALB also Occurs in Untreated, Naïve NSCs although to a Much Lesser Extent

**Figure 5:**
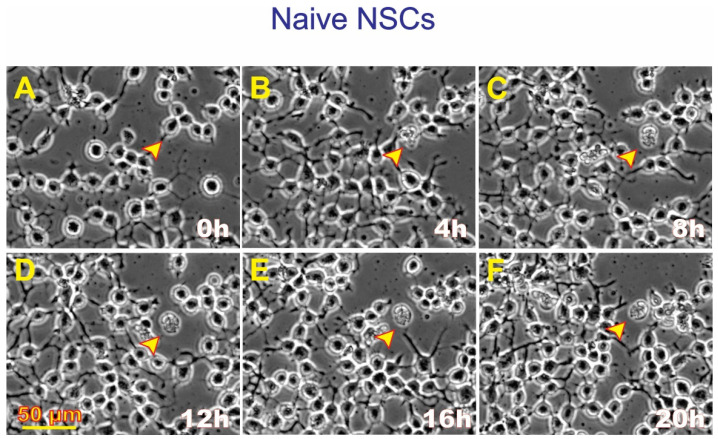
Example of ALB displayed by naïve NSCs. Compared with the naïve cultures incubated with the NSCs’ secretome, it was extremely difficult to locate naïve NSCs presenting ALB in the absence of the space-derived secretome. (**A**) A live cell with two cell processes; (**B**) Four hours later, the cell had excreted some material. (**C**) The cell remained attached to its material. (**D**) No major changes were observed. (**E**) There were no changes. (**F**) Only the debris was observed. This time-lapse sequence below shows the healthy status of the vast majority of naïve NSCs where it was very difficult to find cells undergoing ALB. The process appears to have started between 0 h and 4 h after the time-lapse series had started. The cell remained virtually unchanged after that.

Naïve NSCs were seeded in STMc and kept overnight. The next day, the medium was replaced with freshly prepared STMc medium, and the flaskette was placed in the time-lapse system. The starting time was considered time 0. As an illustration of ALB in naïve NSCs (Figure 5), at 8:20 h, a cell divided and continued moving in various directions. Eventually, the cell expelled intracellular material, re-captured it, and finally died. Therefore, by the 30th hour from the beginning of the time-lapse series, one cell underwent ALB while the second cell remained alive and migratory for the rest of the study.

**Figure 6:**
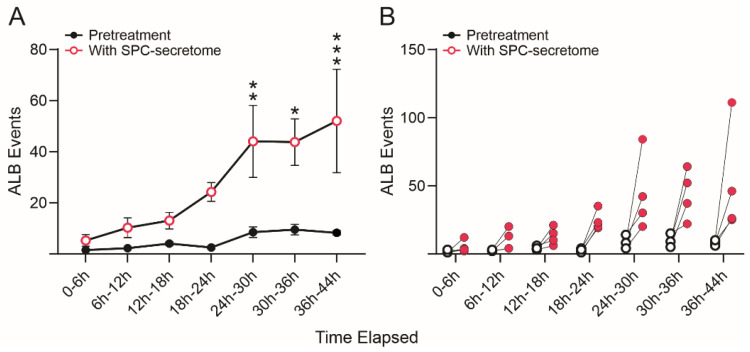
SPC-NSCs-produced secretome enhanced ALB in naïve NSCs. Comparison of the number of ALB events in naïve NSCs in STM medium alone and naïve NSCs grown in medium containing STM + the secretome of SPC-flown NSCs (2:1 *v*/*v*). We found that ALB was upregulated in NSCs treated with the secretome of SPC-NSCs since the beginning of the time-lapse. Untreated naïve NSC cultures had a very low number of cells undergoing ALB starting with 1 to 2 cells in the first 12 h, increasing to an average of 5 to 9 cells until the end of the study 44 h later. (**A**) The graph represents the average number of events observed in the 4 samples examined before and after exposure to the secretome medium. (**B**) The graph shows individual changes in the number of ALB events before (black open dots) and after (red dots) changing to the secretome-containing medium. Statistical analysis was performed using Two-way Repeated Measures ANOVA and the interaction time × pretreatment × secretome was highly significant (*p* = 0.0065, F(6, 18) = 4.418). Tukey’s multiple comparisons post hoc test demonstrated statistically significant increases at the 24–30 h (*p* = 0.0088), 30–36 h (*p* = 0.0123) and (*p* = 0.001) bins. Asterisks indicate the level of statistical significance with * *p* < 0.05, ** *p* < 0.01 and *** *p* < 0.001.

We then compared the frequency of ALB events in naïve NSCs cultured solely in the STM and naïve NSCs grown in a medium containing STM plus the secretome of SPC-flown NSCs (2:1 *v*/*v*). We found that ALB was upregulated in NSCs treated with the secretome of SPC-NSCs since the beginning of the treatment. Naïve NSCs alone had a very low incidence of ALB, no more than once during the first 12 h and no more than 8 to 10 up to 36 h. In contrast, cells with the SPC secretome had their lowest frequency of ALB within the first 6 h. The incidence increased by approximately 5 more events for the next 12 h; 10 more events between 18 h and 24 h, and then it jumped to around 25 events within the following 6 h (from 24 h to 30 h) up to 44 h while untreated naïve NSCs remained at a basal level, as shown in Figure 6.

To eliminate the possibility that the observed effects of the secretome were due to the fact that these cells were derived from hiPS, we next tested the SPC-NSCs’ secretome on naïve non-starved OLPs derived from an 18-week-old human embryonic brain, and we observed a deleterious effect on these OLPs, where they started to die within the first 5 h (Figure S2).

### 3.5. Secretome Analysis

Proteomic analysis of SPC-flown NSCs’ secretome revealed the enrichment of several proteins involved in stress-related pathways [15], and in particular, a subset of proteins associated to processes conducive to ALB. The most significantly enriched members are “Secreted Protein Acidic and Rich in Cysteine” (SPARC, 12.84), Calreticulin (CALR, 5.2), and Endoplasmin (ENPL, 3.3), members of the endoplasmic reticulum (ER) stress response pathway, as well as Heat Shock Protein 90-beta (P90AB1, 7.3), Heat Shock Protein A8 (HSPA8, 3.7), and Vimentin (VIME, 2.1) implicated in chaperone-mediated autophagy and late endosomal microautophagy (Table 1).

## 4. Discussion

Before discussing our findings in more detail, a number of limitations have to be underlined. First, our definition of ALB does not exactly fit the canonical definition of autophagy, namely, the formation of intracellular vacuoles and the degradation of intracellular substrates. Second, due to the nature of the study, experimental manipulations could not be easily performed while cells were subjected to microgravity, e.g., use of traditional autophagy markers such as LC-3. While the size of our sample did not allow us to fully characterize the ALB phenomenon to rule out cell fusion and/or apoptosis, the fact that proteins involved in autophagy were upregulated supports the use of the ALB term as opposed to “apoptosis”. We ascertained that space-flown NSCs had a larger number of ALB events with respect to ground controls, and this is a novel finding that deserves further investigation. Thus, our evidence of ALB is indirect and limited to the effects observed while SPC-NSCs were readapting to Earth’s gravity or effects induced by the secretome of SPCs on control cells. Notwithstanding these limitations, our study provides new insights critical for the understanding of the effects of long-term space travel, including the discovery of SPARC as a potential biomarker of microgravity-induced ALB.

### 4.1. Microgravity as a Potential Modulator of ALB

The lysosomal catabolic degradation mechanism, known as autophagy, is essential to maintain cellular survival and function, and it is activated in situations of cellular stress [16,17]. Autophagy involves autophagosomes that fuse with lysosomes leading to cellular degradation. It has two very critical roles in eukaryotic cells [18,19]: one is nutrient recycling, and the second is eliminating excessive or harmful cellular products such as protein aggregates, supramolecular structures, or organelles such as mitochondria or peroxisomes.

In the present study, one could argue that our cells flown onto space may have been starved because they were unattended, and that the ALB observed was a direct consequence of this. Nonetheless, when ground control cells were grown on Earth’s gravity in the same conditions, ALB remained within basal levels throughout the experiment. Moreover, the naïve NSCs were not deprived of nutrients at any point in time; and yet, those receiving fresh culture medium supplemented with the SPC-NSC secretome displayed ALB from the beginning of the experiment and the frequency of events increased with time spent in contact with the space secretome. Thus, the secretome effect was much more potent than that of space flight. Since these cells responded to the secretome in 1 G and without starvation, it is likely that this phenomenon was upregulated via one or more of the secreted proteins. Thus, SPARC appears to be a gravity sensor whose effects are potentiated by Earth’s gravity and mitigated while in microgravity. Yet, instead of triggering an effect similar to that on SPC-flown NSCs and naïve NSCs, the SPC-flown secretome was very deleterious to OLPs derived from an embryonic brain, indicating that OLPs are more vulnerable to the SPC-produced secretome than NSCs. Studies on pathways to cell death have shown that rapamycin induces starvation-like behaviors by blocking mTOR [20]. Chaperone-mediated autophagy was the first studied process indicating degradation of intracellular components by the lysosome. It is a selective autophagic pathway mediated by chaperones such as HSPA8 [20]. In contrast, late endosomal microautophagy is a non-selective autophagic pathway that involves internalization of cytosolic cargo through invagination of the lysosomal membrane [21]. Microautophagy is coordinated and complements other forms of self-eating pathways, such as chaperone-mediated autophagy and macroautophagy, among others [22].

Considering this is a pioneer study, the scope of our grant did not include an in-depth molecular characterization because neither sample size nor funding would allow it. Nonetheless, space flight definitively exerts stress on mammalian cells and organisms, which most likely contributed to ALB described here.

### 4.2. Microgravity as a Modulator of ALB Proteins

Space flight involves a plethora of stressful events for individuals and cells in culture. Examination of the secretome of NSCs after space flight showed an increase in SPARC, which is known to participate in extracellular matrix synthesis and remodeling as it intervenes in the morphological changes of cells. SPARC is also known to be present during embryonic and postnatal growth in radial glia cells, blood vessels, and structures originating from the pia [23]. The rostral migratory stream also displays this protein, and its sequential spatial restriction leads to its expression solely in the adult’s brain sub-ventricular zone (SVZ). In specialized glial cells, SPARC expression is enhanced [23]. Exogenous SPARC stimulates cell growth in low serum correlating with metastatic behavior [24,25,26,27]. Plasmid-overexpressed SPARC also triggers stress of the ER and unfolded protein response (UPR). Interestingly, inhibition of ER stress leads to an inhibition of autophagy-mediated apoptosis. Thus, ER stress plays a critical role in the regulation of autophagy-mediated apoptosis in SPARC-overexpressing neuroblastoma cells and radiation therapy [28,29]. Taken together, these studies suggest that our NSCs that were grown in a culture medium without serum and in space may have acquired characteristics approaching those of cancerous cells insofar as they proliferated more than sister cultures grown on Earth [4]. In addition, its expression increases in models of glutamate excitotoxicity and when knocked down, reduces neuronal injury [30]. The same study showed that glutamate excitotoxicity involves autophagy. Upon glutamic acid stimulation, LC3II/LC3I increases resulting in autophagy activation [30]. Overexpression of SPARC induces apoptosis in medulloblastoma by triggering ER stress and UPR [29]; and in neuroprimitive neuroectodermal tumors (PNET) through different pathways [29,31,32]. Vimentin regulates numerous cellular processes including signal transduction, and the spatiotemporal organization of organelles for proper cell function. It has been demonstrated that inhibition of vimentin results in its aggregation leading to an accumulation of autophagosomes, consequently interfering with their fusion with lysosomes due to modulation of the mechanistic target of rapamycin (mTORC1) pathway [33]. In addition, an emergent field of study is being developed aiming to elucidate a possible link between the cytoskeleton and autophagy [33,34]. Vimentin was also upregulated by microgravity, supporting the idea that it might also contribute to ALB.

Although usually recognized as an intracellular protein, the secretion of vimentin by astrocytes has been reported where it interacts with insulin-like growth factor 1 receptor (IGF1R) resulting in axonal growth [35]. The authors found that there is an interaction of both vimentin and IGF1 and IGF1R, which directly bind to vimentin, through residues number 330–407. In addition, vimentin is overexpressed and secreted by endothelial cells using the type III unconventional mechanism, and it mimics vascular endothelial growth factor (VEGF) as a pro-angiogenic factor [36]. Although less common, vimentin can be found in the extracellular culture medium. It also regulates NSCs’ quiescence and protein clearance in the brain. Hence, vimentin plays important roles in brain homeostasis.

Recently, studies in culture demonstrated three important facts: (i) that the SARS-CoV-2 receptor binding domain (RBD) surface vimentin increases the invasive potential of non-tumorigenic (MCF-10a) and cancer (MCF-7) cells; (ii) that added recombinant vimentin binds to the cell surface, enhancing the permeability of monolayers of both cell lines MCF-10a and MCF-7 to the virus; (iii) that when adding SARS-CoV-2 receptor binding domain, extracellular vimentin directly binds to it and, therefore, the permeability effects on MCF-7 are lost [37]. Moreover, vimentin also exists in the extracellular matrix, on the surface of the cell’s cytoplasmic membrane. Previous studies have shown that vimentin may exert multiple physiological effects in different nervous system injuries and diseases. For example, studies with knockout mice have shown that its modulation makes it an interesting target to attenuate infection, inhibit progression of brain tumors and to enhance axonal myelination [38]. Thus, more unusual roles of extracellular vimentin are being discovered.

The above examples were all generated on Earth’s gravity. Our novel data derive from cells grown in microgravity, and these findings where NSCs secreted vimentin in microgravity were surprising. More research needs to be conducted to characterize new roles of vimentin in space as well as on Earth. Therefore, future studies need to include this protein as part of the analysis of cells flown to space.

Modulation of SPARC on Earth appears to be an appealing approach to arrest the growth of cancerous cells in several organs and cell types [30,39]. Nonetheless, it is possible that its aberrant elevated expression promoted by microgravity induces increased NSCs proliferation [4]. Moreover, as shown in the present study, it may be deleterious if left uncontrolled in the brain of astronauts embarking on long-term space travel such as to the Moon and Mars. Because SPARC plays many roles in the fate and physiology of cells, it is plausible that it is a target protein whose modulation will help maintain cellular homeostasis in the brain. Regulation and maintenance of healthy levels of SPARC and cell numbers in the CNS are of utmost importance for the success of long-term space missions. By suppressing the elevated expression of SPARC, we may be able to reduce the excessive proliferation of NSCs that occurs in cases like that of melanoma cells, where SPARC-acquired expression increases their survival by suppressing p53 and the subsequent inhibition of the apoptotic pathway [40].

Based on our results, we consider SPARC as a microgravity signature of important gravitationally regulated changes that deserve further elucidation to determine if the type of phenomenon observed is cell fusion or autophagy. At the present time, we can assign to SPARC the term of “biomarker” for ALB. More studies using microgravity will allow the characterization of all the proteins that might have contributed to the deleterious ALB in our cells.

### 4.3. Space Flight and Radiation during SpX-16

Among other potential contributing factors for the phenomena observed is radiation. There are two sources of ionizing radiation onboard the ISS, galactic cosmic radiation (GCR) and trapped protons whose dose accumulates mainly during passages through the South Atlantic Anomaly (SAA). The instruments that took the data from the Space Radiation Analysis Group, Johnson Space Center (JSC-SRAG), use information on the ISS orbit in combination with mapping the geomagnetic field to break down the dose into GCR and SAA components. The actual radiation during the space mission is shown in Table 2.

Although in the present manuscript we did not address the effects of radiation on NSCs while in space, the average dose of radiation is 0.3–0.4 mSv/day in ISS, where protons are the principal space radiation source in the ISS. Therefore, SPC-NSCs were exposed to some radiation, adding to the stress of their ER.

Because autophagy affects cell proliferation and survival, reports have shown that modulation of autophagy can improve the outcome of cancer treatment combined with radiotherapy [41]. For glioblastoma, the most aggressive brain cancer, Palumbo and collaborators [42] described a differential involvement of autophagy in two human malignant glioma cell lines undergoing temozolomide and radiation treatments combined. In such a model, radiation-induced ER stress protein expression is linked to protein folding, UPR, and radiation-induced autophagy [43]. The suppression of autophagy is now accepted as an important cytoprotective mechanism that enhances the response of cancer cells to multiple treatments [44]. Lastly, it has been reported that TNFalpha produces a similar pattern of morphological changes including bleb-formation in their cytoplasmic membrane, shrinkage, and death. Nonetheless, this type of cell death is not autophagy-related. In cases of inflammatory processes, blebbing of cell membranes occurs in order to phagocyte, clear, and prevent inflammatory/autoimmune response [45].

In our case, the therapeutic targeting of ALB in NSCs may be the key to mitigating intracranial hypertension in astronauts, and radiation deserves deeper investigation as a subject with the aim of producing combinatorial therapies for the successful future of space exploration.

## 5. Conclusions

Here we report two novel findings: the increased secretion of ER stress related proteins SPARC, calreticulin, and endoplasmin by space flight and the induction of ALB in NSCs detected after space flight. While the SPC-NSCs-produced secretome gave rise to the same effects on naïve NSCs, we have also observed a similar phenomenon produced by the secretome of SPC-flown OLPs [46]. Nonetheless, the different cell types that form the CNS and the vasculature, either alone or in concert, may respond differently to microgravity. Therefore, it is important to examine proteogenic upregulation in the context of the entire brain during, prior to, and after space flight, where all the cell types interact and work in concert, leaving the possibility that one cell type may prevent SPARC up-regulation and ALB.

One question that comes to mind is that while in space, the exposure to microgravity also implies exposure to radiation. We believe that this is a promising start that will allow us to address intracranial hypertension in astronauts, preventive measures for future astronauts flying to space as well as effective countermeasures for astronauts currently in the ISS and those that have preceded them.

## Figures and Tables

**Table 1:** Enrichment of the top-ranked proteins.

	Proteomic Profile of SPC-NSCs-Produced Secretome		
SPC/1G	Accession No.	Gene	Top Ranked Protein (Species)
12.84	HUMAN	SPARC	SPARC OS = Homo Sapiens OX = 9606 GN = SPARC PE = 1 SV = 1
7.3	HUMAN	P90AB1	Heat shock protein HSP 90-beta OS = Homo sapiens OX = 9606 GN = HSP90AB1 PE = 1 SV = 4
5.2	HUMAN	CALR	Calreticulin OS = Homo sapiens OX = 9606 GN = CALR PE = 1 SV = 1
3.7	HUMAN	HSPA8	Heat shock cognate 71 kDa protein OS = Homo sapiens OX = 9606 GN = HSPA8 PE = 1 SV = 1
3.3	HUMAN	ENPL	Endoplasmin OS = Homo sapiens OX-9606 GN = HSP90B1 PE = 1 SV = 1
3.0	HUMAN		Vimentin OS = Homo sapiens OX = 9606 GN = VIM PE = 1 SV = 4

**Table 2:** Radiation information for the space mission.

	Mission GCR Dose (mGy)	Mission SAA Dose (mGy)	Mission Total Dose (mGy)	Maximum Daily Total Dose (mGy)	Minimum Daily Total Dose (mGy)	Average Daily Total Dose (mGy)
SpX-16	5.197	8.022	13.219	0.417	0.322	0.357 (0.026)
				(12/17/18)	(1/12/19)	

## Data Availability

The data presented in this study are available on request from the corresponding author.

## Supplementary Materials

The following supporting information can be downloaded at: https://www.mdpi.com/article/10.3390/biom14010065/s1, Figure S1: Spaceflight automated experiment, Figure S2: The Secretome of SPC-Flown Neural Stem Cells is deleterious to Naïve OLPs, Video S1: SPC-NSCs autophagy two weeks postflight, Video S2: View of Naïve NSCs with NSCs secretome, Video S3: High magnification view of naïve human OLPs with NSCs secretome, Table S1: List of abbreviations and their meaning. Reference [12] is cited in the Supplementary Materials.
