# Supplementary material for: Space Flight Enhances Stress Pathways in Human Neural Stem Cells
Source: Biomolecules. 2024 Jan 3;14(1):65. doi: 10.3390/biom14010065 (PMC10813251; doi:10.3390/biom14010065)
Supplement: Supplementary file 1 [file biomolecules-14-00065-s001.zip › 240101_SupplementalFigs.pdf]

Supplemental Materials

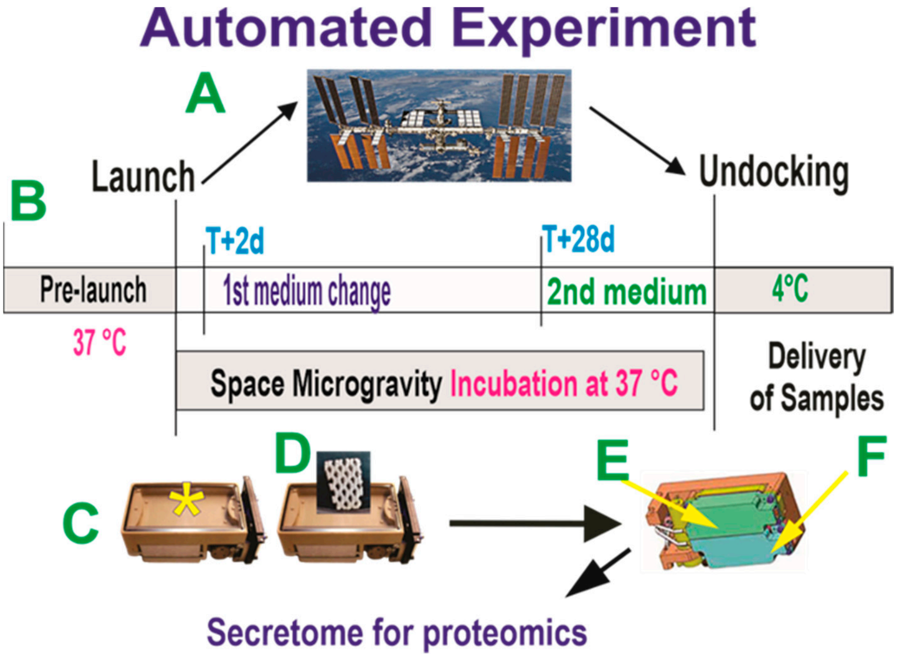

Figure S1. Spaceflight automated experiment. As part of the Bioscience-4 Space Biology NASA experiment, flew to the ISS on board of SpaceX-16. The cells remained in microgravity for approximately 39.3 days, on board the International Space Station in an orbit of 354 miles above Earth. A) View of the ISS. B) Shows the timeline for the experiment. C) View of the automated unit, the asterisk shows the chamber where cells were seeded and cultured. D) Shows a view of the mesh carriers. E,F) Bottom view of the Type IV unit showing the two tanks. Tank 1 medium is used for the 1st media change and Tank 2 medium was pumped into the cell chamber during the 2nd media change. Following 2nd media change, the units were stored at 4°C until return to Earth.

Cultures of Naïve human embryonic brain OLPs (Method for OLPs)

We used OLPs derived from human embryonic brain and cultured them in OLBN, a chemically defined medium designed for the propagation and maintenance of OLPs (Espinosa et al., 2002; 2009, 2016). Human cortical tissue samples were de-identified and voluntarily donated; aborted specimen remains pre-shelved in Pathology and thereby Institutional Review Board exempt. Neural progenitors were purified from a cell suspension of human fetal cortex at 15–17 weeks of gestation using a percoll gradient [10]. The volume of the filtered cell suspension in growth medium (DMEM/F12, 10% FBS, 1:1000 gentamicin) was determined and a final 30% percoll solution was made by mixing one part of a HBSS-buffered percoll solution to two parts of cell suspension. The mixture was centrifuged in Oakridge tubes at 30,000 g at 4°C for 30 min. The bottom half fraction was transferred to a new 50 ml tube and the pellet of blood cells was discarded. Then, the percoll suspension was diluted in two volumes of growth medium and the cells were collected by centrifugation at 400 g for 10 min. The cell pellet was resuspended with 20 ml of growth medium per tube and plated onto uncoated 100 mm non-adherent

petri dishes. After 48 h, the cell suspension from two dishes was transferred into a 50 ml tube and centrifuged for 5 min at 400 g, next the cells were resuspended in 24 ml of fresh growth medium and plated in 75 mm tissue culture flasks (12 ml/flasks). On the next day, 6 ml of growth medium was replaced with oligodendrocyte specification medium (OSM) to induce oligodendrocyte lineage commitment. This last step was repeated every four days until obtaining the desired cell density. After reaching confluency, cells were mechanically detached and replated in OSM either in 12.5 mm flasks or glass flaskettes. After 72 h half of the cultures were placed in the 3D-Clinostat and half cultures were kept in the same incubator in 1G. To ascertain the effects of SPC-secretome on naïve OLPs, we used OLBN and SPC-NSCs secretome in a ratio of 2:1 respectively.

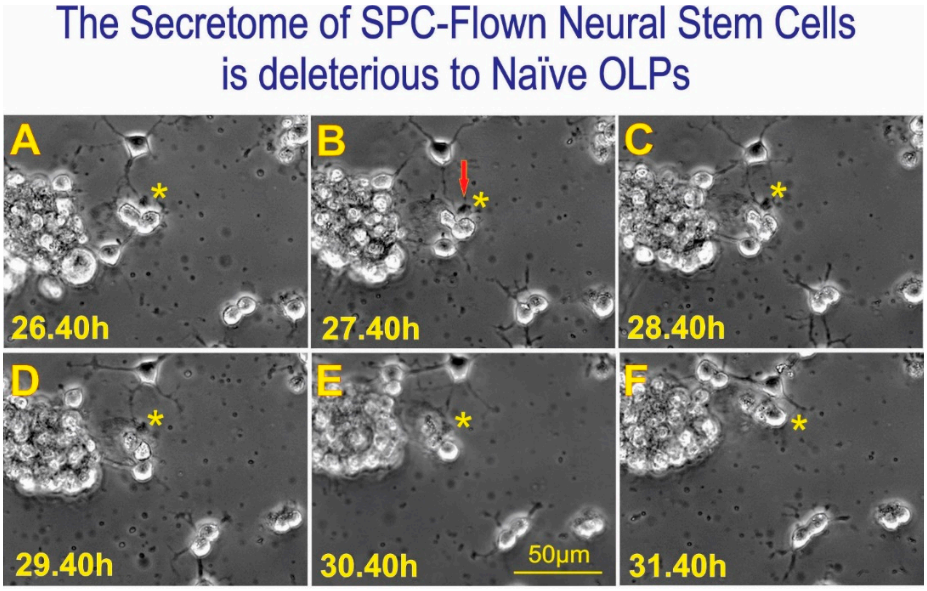

Figure S2. Naïve human OLPs were seeded and cultured onto Poly-d-Lysine coated flaskettes and fed with OLBN medium (Espinosa et al., 2016). The following day the culture medium was replaced with fresh OLBN and SPC-NSCs secretome (2:1 V/V respectively) and placed in the time lapse system. A) Overview of the initial frame showing the condition of the OLPs just after the medium was added to the cultures. Most if not all cells displayed two or multiple cell processes. B) One day after treatment, most OLPs were devoid of cell processes, they were surrounded by debris and had started to die. C) Compared with naïve NSCs, naïve OLPs incubated with the NSCs' secretome appeared to be more sensitive to the treatment. In spite of that, some cells were still trying to divide.

Table S1. List of abbreviations and their meaning.

List of Abbreviations	Meaning
ALB	Autophagy-like behavior
CALR	Calreticulin
CNS	Central nervous system
ENPL	Endoplasmin
ER	Endoplasmic reticulum

GC	Ground control
GCR	Galactic cosmic radiation
hiPS	Human induced pluripotent stem cells
HSPA8	Heat Shock Protein A8
IEF	Isoelectric focusin
IPG	Immobilized pH gradient
IRB	Institutional review board
ISS	International Space Station
JSC-SRAG	Space Radiation Analysis Group, Johnson Space Center
MALDI	Matrix assisted laser desorption/ionization
MALDI-TOF MS	Matrix assisted laser desorption/ionization-time of flight mass spectrometry
MS	Mass spectrometry
NCBInr	National Center for Biotechnology Information non-redundant
NIH	National Institutes of Health
NSC	Neural stem cell
OLP	Oligodendrocyte progenitors
P90AB1	Heat Shock Protein 90-beta
PNET	Neuroprimitive neuroectodermal tumors
SAA	South Atlantic Anomaly
SDS	Sodium dodecyl-sulfate
SDS-PAGE	Sodium dodecyl-sulfate polyacrylamide gel electrophoresis
SPARC	Secreted protein acidic and rich in cysteine

Video S1. SPC-NSCs exhibited autophagy-like behavior (ALB) two weeks post-flight. This time-lapse sequence shows the conditions of the cells when the time-lapse capture started. They were arranged as single cells or clusters made-up of clonal cell proliferation. The cells look healthy and bear multiple cell processes. The yellow arrow shows a cell that slowly deteriorated and died by autophagic cell death. This mp4 video is displayed at 4 frames per second, where frames represent image captures taken 10 minutes apart.

Video S2. View of Naïve NSCs with space-flown NSCs secretome. This time-lapse sequence shows the condition of the cells when the time-lapse capture started. They all bore cell processes and were arranged as single cells or clusters made-up of clonal cell proliferation. The dynamics of an autophagy-like event wherein the cell was bipolar and in contact with two other cells held by each cell process, eventually the cell bore the morphology of a small dividing cell and held by opposite cells as trying to help it divide. Nonetheless it did not divide and excreted some material moving among neighboring cells and finally dying by the end of the timelapse. The culture aspect had changed enormously as many NSCs proliferated giving rise to more and larger clusters. This mp4 video is displayed at 4 frames per second, where frames represent image captures taken 10 minutes apart.

Video S3. View of naïve human OLPs with NSCs secretome. In order to test if the effects of SPC-NSCs secretome were cell-specific related, we added it to human OLPs that had not been exposed to microgravity. The majority of these cells, whether as clusters or as single cells, had become unhealthy with ruffle-like cytoplasmic membranes, most of them were devoid of cell processes, and their debris were visible on the surface of the flaskette. The arrow points to a cell that is located near unhealthy or dead cells. This particular cell, although unhealthy, still bore short processes nonetheless and at a given point collapsed depositing all its intracellular material onto the sub-stratum. (For details of the experiment, see main text). This mp4 video is displayed at 4 frames per second, where frames represent image captures taken 10 minutes apart.
