# Supplementary figures and images for: Space Flight Enhances Stress Pathways in Human Neural Stem Cells
Source: Biomolecules. 2024 Jan 3;14(1):65. doi: 10.3390/biom14010065 (PMC10813251; doi:10.3390/biom14010065)

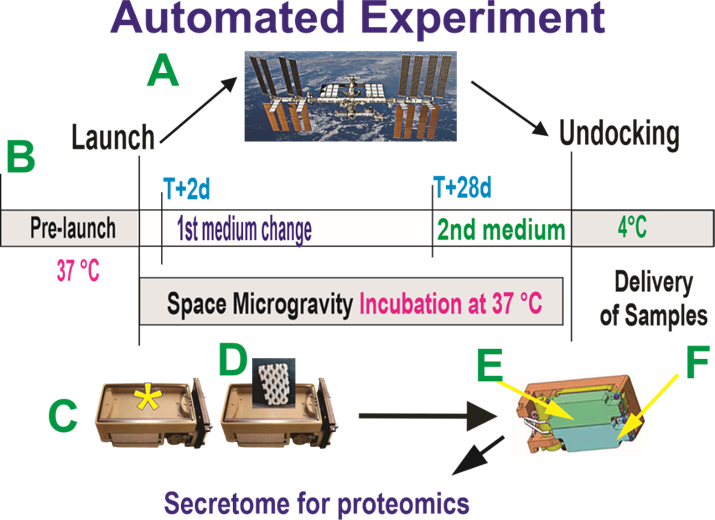

Supplement: Supplementary file 1 [file biomolecules-14-00065-s001.zip › FigS1_Spaceflight_automated_experiment.tif]

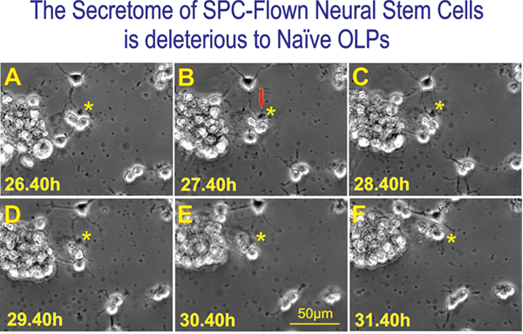

Supplement: Supplementary file 1 [file biomolecules-14-00065-s001.zip › FigS2_Naive_OLPs_w_SPCSecretom.tif]
